# Clinical Characteristics, Prognosis, and Gender Disparities in Young Patients With Acute Myocardial Infarction

**DOI:** 10.3389/fcvm.2021.720378

**Published:** 2021-08-12

**Authors:** Junxing Lv, Lin Ni, Kexin Liu, Xiaojin Gao, Jingang Yang, Xuan Zhang, Yunqing Ye, Qiuting Dong, Rui Fu, Hui Sun, Xinxin Yan, Yanyan Zhao, Yang Wang, Yuejin Yang, Haiyan Xu

**Affiliations:** ^1^Department of Cardiology, Fuwai Hospital, National Center for Cardiovascular Diseases, Chinese Academy of Medical Sciences and Peking Union Medical College, Beijing, China; ^2^Medical Research & Biometrics Center, Fuwai Hospital, National Center for Cardiovascular Diseases, Chinese Academy of Medical Sciences and Peking Union Medical College, Beijing, China

**Keywords:** myocardial infarction, percutaneous coronary intervention, young patient, gender, prognosis

## Abstract

**Background:** Young people hold a stable or increasing percentage of patients with acute myocardial infarction (AMI) in many countries. However, data on clinical characteristics and outcomes of young AMI patients were insufficient. This study aimed to analyze clinical characteristics, prognosis, and gender disparities in patients aged ≤45 years with AMI.

**Methods:** A total of 24,125 patients from China Acute Myocardial Infarction registry were included in this study. Clinical characteristics, managements, and in-hospital and 2-year outcomes were compared between patients aged ≤45 years and those aged >45 years. Predictors of all-cause death were obtained using multivariate regression models. Gender disparities of AMI were analyzed among young patients.

**Results:** Of 24,125 patients, 2,042 (8.5%, 116 female) were aged ≤45 years. Compared with patients aged >45 years, young patients were more often male, current smokers, and more likely to have medical history of hyperlipidemia. Smoking (72.1%) was the major modifiable risk factor in patients aged ≤45 years. Young patients received more evidence-based medications and had significantly lower risk of both in-hospital and 2-year adverse events than older patients. Education level and left ventricular ejection fraction were independent predictors of 2-year mortality in young patients. Moreover, symptom onset to admission time of young women was significantly longer than that of young men. Young women were less likely to receive percutaneous coronary intervention and suffered higher risk of in-hospital adverse events than young men (adjusted odds ratio for death: 5.767, 95% confidence interval 1.580–21.049, *p* = 0.0080; adjusted odds ratio for the composite of death, re-infarction, and stroke: 3.981, 95% confidence interval 1.150–13.784, *p* = 0.0292). Young women who survived at discharge had a higher 2-year cumulative incidence of death (3.8 vs 1.4%, *p*_log−rank_ = 0.0412).

**Conclusions:** Patients aged ≤45 years constituted a non-negligible proportion of AMI patients, with higher prevalence of smoking and hyperlipidemia but better care and prognosis compared with older patients. There were significant gender disparities of managements and outcomes in young patients. More efforts to improve quality of care in young women are needed.

## Introduction

Although the incidence of acute myocardial infarction (AMI) has been decreasing in developed countries (1, 2), AMI remains one of the main cardiovascular diseases worldwide. Due to the low incidence of AMI in young adults, patients with premature AMI were overlooked in many clinical trials. However, data showed that young people held a stable or increasing percentage of AMI hospitalizations in many countries (3–5). A series of studies have observed different cardiovascular risk factor profiles and better prognosis in young patients compared with aged patients (6, 7). Nevertheless, most studies were limited by very small number of young AMI patients. In this subgroup of patients, conflicting results of gender differences exist and predictors of mortality are still unclear. Moreover, research on prognosis of young AMI patients mainly focused on survival outcome (8). Other clinical endpoints, such as readmission and medication use, are equally important because of the long survival expectation of young patients.

As the largest developing country in the world, China bears a heavy burden of cardiovascular diseases. From 2002 to 2017, the mortality of AMI in China has been increasing annually (9). A similar trend was found in hospitalization rate of young AMI patients (10). However, very few studies focused on young AMI patients in China (5, 11). The study on these patients may have novel implications for understanding the status and quality of AMI management.

Using database of the China Acute Myocardial Infarction (CAMI) registry, this study compared clinical characteristics, treatment, in-hospital and 2-year outcomes between patients aged ≤45 years and those aged >45 years, and identified the predictors of in-hospital and 2-year mortality. Due to the particularity of young women with AMI, we further analyzed gender disparity of patients' characteristics, treatment, and prognosis in young group.

## Methods

### Study Population

Details about the CAMI registry has been published elsewhere (12). In brief, this is a nationwide, multicenter, prospective observational study enrolling 108 hospitals that cover 27 provinces and four municipalities throughout Mainland China. These hospitals were instructed to enroll AMI patients consecutively and the final diagnosis must meet the third Universal Definition for Myocardial Infarction (2012) (13). Presenting features, risk factors, medical history, in-hospital medications, and clinical outcomes were collected by trained clinical cardiologist or cardiovascular fellows. Variables were defined according to the ACC/AHA Task Force on clinical data standards and NCDR-ACTION-GWTG element dictionary.

**Figure 1:**
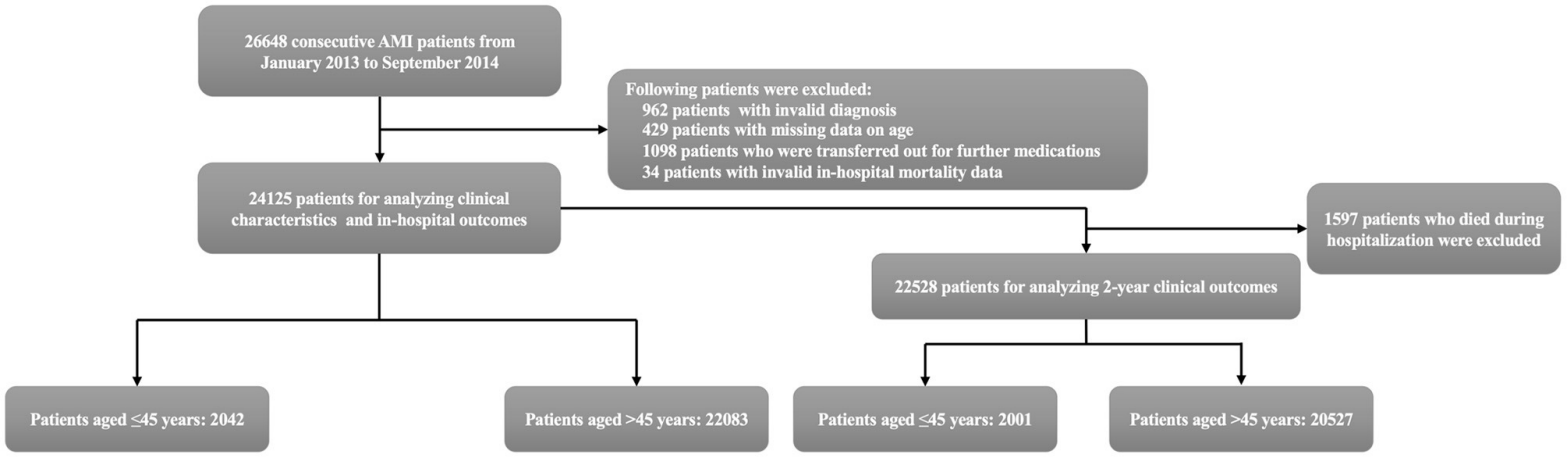
Flowchart of this study. AMI, acute myocardial infarction.

Patients between January 2013 and September 2014 in the registry were included in this analysis. The following patients were excluded: patients with invalid diagnosis (*n* = 962); patients with missing data on age (*n* = 429); patients who were transferred out for further medications (*n* = 1,098); patients with invalid in-hospital mortality data (*n* = 34). Analyses of patients' characteristics and in-hospital outcomes were based on remaining 24,125 patients. After further excluding patients who died during hospitalization, the cohort for evaluating 2-year outcomes consisted of 22,528 patients. Patients were stratified into two age groups (≤45 and >45 years), as the main objective of this analysis was to assess clinical characteristics and outcomes in young AMI patients (Figure 1). To further explore gender disparity in young patients, those aged ≤45 years were divided by sex.

### Follow-Up and Endpoints

Patients who survived to hospital discharge were followed by telephone call or clinical visit at 30 days, 6 months, 12 months, 18 months, and 24 months. Clinical outcomes, including death, recurrent myocardial infarction, stroke, rehospitalization for heart failure, and medication use, were collected. Two-year follow-up rate of patients who survived to hospital discharge was 95.6%.

The primary endpoint of this analysis was all-cause death during hospitalization and at 24 months. The second endpoints included major adverse cardiac and cerebrovascular events (MACCE), all-cause readmission, rehospitalization for heart failure, and medication use at different time points. MACCE were defined as the composite of death, recurrent myocardial infarction, and stroke. Dates of readmission and rehospitalization for heart failure were not documented in CAMI registry, so they were not included in MACCE. Due to high missing rates of medication use after 1-year follow-up, only medications at 30 days, 6 months, and 12 months were taken into account.

### Statistical Analysis

According to data distribution, continuous variables were given as mean ± SD or medians (quartiles) and compared by Student *t* test or non-parametric statistical test. Categorical variables were reported as frequencies (percents) and compared by likelihood-ratio χ^2^ or the Fisher exact test, as appropriate. The cumulative incidences of all-cause death and MACCE were estimated by Kaplan–Meier method. Differences between curves were evaluated by log-rank test.

We used logistic and Cox regression models to analyze the impact of age (as a categorical variable) on in-hospital and 2-year clinical outcomes, respectively. The logistic regression models for in-hospital outcomes were adjusted as follows: sex, medical insurance, education level, living condition, body mass index (BMI), diabetes mellitus, hypertension, hyperlipidemia, smoking status, prior angina pectoris, prior myocardial infarction, prior heart failure, prior stroke, prior percutaneous coronary intervention (PCI), prior renal dysfunction, chronic obstructive pulmonary disease (COPD), family history of premature coronary atherosclerosis heart disease, symptom onset to admission time, heart rate, systolic blood pressure, Killip grade, cardiac arrest at admission, diagnosis, anterior wall involvement, left ventricular ejection fraction (LVEF), creatinine level, hemoglobin and leukocyte count. Besides these covariates, the Cox regression models for 2-year outcomes were further adjusted by PCI and medications at discharge.

Furthermore, to explore predictors of in-hospital and 2-year all-cause death in patients aged ≤45 years, univariate logistic and Cox models were first constructed. Age and other variables with *p* < 0.25 in univariate analysis were chosen to fit the multivariate models with backward selection method. As a potential predictor of death, age was analyzed as a continuous variable in this part of the study.

In addition, we particularly examined gender disparity in patients aged ≤45 years. Six adjusted models were conducted to find out a clear explanation for differences in rates of PCI between men and women. Logistic and Cox regression models were performed to analyze the impact of sex on in-hospital and 2-year clinical outcomes. Besides taking age as a continuous variable, similar covariates were adjusted as mentioned previously. More details of analysis on gender disparities in young patients are shown in Supplementary Material. We used variance inflation factor to examine multicollinearity between variables before being entered into multivariate regression models. Results from multivariate models were expressed using odds ratios (OR) or hazard ratios (HR) with 95% confidence intervals (CI), as appropriate. Two-tailed *p* < 0.05 was considered statistically significant. All statistical analyses were performed using SAS version 9.4 (SAS Institute Inc., Cary, NC, USA).

## Results

### Patients in the Analysis

Among 24,125 patients in the cohort for analyzing clinical characteristics and in-hospital outcomes, 2,042 (8.5%) patients were no more than 45 years old. Among 22,528 patients in the cohort for evaluating 2-year outcomes, 2,001 (8.9%) were at or under 45 years old (Figure 1).

### Baseline Characteristics

Baseline characteristics are presented in Table 1. The mean age in two groups were 39.95 ± 4.64 and 65.06 ± 10.70 years, respectively (*p* < 0.0001). Compared with patients aged >45 years, young patients were more often male, current smokers, and more likely to have a medical history of hyperlipidemia and a family history of premature coronary artery disease. The percentages of patients with BMI ≥28 kg/m^2^, diabetes mellitus, hypertension, hyperlipidemia, and current smoking were 18.3, 11.3, 33.5, 10.4, and 72.1% in patients aged ≤45 years, respectively, and 1,740 (85.2%) patients had at least one of these five risk factors. The prevalence of diabetes mellitus, hypertension, prior angina pectoris, prior myocardial infarction, prior heart failure, prior stroke, and COPD in young patients were significantly lower than those in older patients. There were no significant differences in the proportion of asymptomatic patients between groups. However, compared with patients over 45 years old, symptom onset to admission time of young patients was significantly shorter. Younger patients more frequently presented with ST-segment elevation and had anterior wall involvement than older patients.

**Table 1:** Baseline characteristics of patients in different age groups.

**Variables**	**Patients aged ≤45 years**	**Patients aged >45 years**	***P*-value**
	**(*n* = 2,042)**	**(*n* = 22,083)**	
Patient demographics			
Age, years	39.95 ± 4.64	65.06 ± 10.70	<0.0001
Female	116 (5.7)	6058 (27.4)	<0.0001
Self-paying medications	245 (12.8)	1,579 (7.6)	<0.0001
Education level			<0.0001
University/college or higher	316 (21.5)	1,597 (10.4)	
Secondary school	856 (58.3)	6,106 (39.8)	
Primary school or illiterate	296 (20.2)	7,631 (49.8)	
Living alone	69 (3.4)	628 (2.9)	0.1767
BMI	25.44 ± 3.41	23.97 ± 3.09	<0.0001
BMI ≥28 kg/m^2^	360 (18.3)	1,731 (8.2)	<0.0001
Medical history			
Diabetes mellitus	220 (11.3)	4,357 (20.8)	<0.0001
Hypertension	662 (33.5)	11,366 (53.1)	<0.0001
Hyperlipidemia	188 (10.4)	1,496 (7.9)	0.0004
Current smoker	1,449 (72.1)	8,951 (41.3)	<0.0001
Prior angina pectoris	354 (18.5)	5,873 (29.0)	<0.0001
Prior myocardial infarction	87 (4.5)	1,610 (7.9)	<0.0001
Prior heart failure	18 (0.9)	558 (2.7)	<0.0001
Prior stroke	47 (2.4)	2,148 (10.2)	<0.0001
Prior PCI	62 (3.2)	1,069 (5.1)	<0.0001
Prior renal dysfunction	14 (0.7)	301 (1.4)	0.0037
COPD	5 (0.3)	459 (2.2)	<0.0001
Family history of premature CAD	124 (7.1)	672 (3.8)	<0.0001
Presenting characteristics			
Symptom			0.2869
Yes	2,008 (99.6)	21,561 (99.4)	
No	9 (0.4)	137 (0.6)	
Trigger factor			<0.0001
Yes	494 (26.7)	4,142 (21.1)	
No	1,354 (73.3)	15,518 (78.9)	
Symptom onset to admission time			<0.0001
<3 h	563 (27.7)	4,632 (21.2)	
3–6 h	524 (25.8)	5,267 (24.1)	
6–12 h	324 (15.9)	3,545 (16.2)	
12–24 h	228 (11.2)	2,450 (11.2)	
1–7 days	395 (19.4)	5,933 (27.2)	
Heart rate	80.17 ± 16.38	77.92 ± 19.13	<0.0001
Systolic blood pressure	128.47 ± 22.55	128.76 ± 25.89	0.5880
Killip grade			<0.0001
I	1,742 (86.8)	15,821 (73.0)	
II	196 (9.8)	3,810 (17.6)	
III	32 (1.6)	1,124 (5.2)	
IV	38 (1.9)	930 (4.3)	
Cardiac arrest at admission	25 (1.2)	256 (1.2)	0.7980
Diagnosis			<0.0001
STEMI	1,727 (84.6)	16,533 (74.9)	
NSTEMI	315 (15.4)	5,550 (25.1)	
Anterior wall involvement	1,053 (52.5)	10,356 (47.8)	<0.0001
Laboratory results			
Creatinine, μmol/L	71.40 (61.00, 83.10)	75.60 (62.30, 93.00)	<0.0001
Hemoglobin, g/L	149.52 ± 19.05	134.41 ± 21.38	<0.0001
Leukocyte count, × 10^9^/L	11.52 ± 4.01	10.06 ± 3.71	<0.0001
LVEF, %	55.00 ± 10.33	53.28 ± 10.97	<0.0001

*BMI, body mass index; PCI, percutaneous coronary intervention; COPD, chronic obstructive pulmonary disease; CAD, coronary artery disease; STEMI, ST-segment elevation myocardial infarction; NSTEMI, non-ST-segment elevation myocardial infarction; LVEF, left ventricular ejection fraction. Data are reported as n (%), mean ± SD or median (quartiles)*.

### Angiographic Characteristics of Patients Undergoing Primary PCI

Of 24,125 patients, 8,700 (36.1%) underwent primary PCI. Angiographic characteristics are summarized in Table 2. In brief, transradial approach was more frequently used in patients aged ≤45 years than in patients aged >45 years. Compared with older patients, young patients were more likely to suffer left anterior descending artery related myocardial infarction. The rates of stent implantation, and pre- and post-procedural TIMI flow grade were similar in two groups. Similar results were found in STEMI patients undergoing primary PCI (Supplementary Table 1).

**Table 2:** Angiographic characteristics of patients undergoing primary PCI in different age groups.

**Variables**	**Patients aged ≤45 years**	**Patients aged >45 years**	***P*-value**
	**(*n* = 943)**	**(*n* = 7,757)**	
Diagnosis			0.0018
STEMI	903 (95.8)	7,233 (93.2)	
NSTEMI	40 (4.2)	524 (6.8)	
Transradial approach	806 (91.0)	6,134 (86.8)	0.0003
Culprit artery			0.0003
Left anterior descending artery	510 (55.4)	3,574 (47.7)	
Left circumflex artery	99 (10.8)	886 (11.8)	
Right coronary artery	289 (31.4)	2,790 (37.3)	
Left main coronary artery	4 (0.4)	68 (0.9)	
Others	18 (2.0)	167 (2.2)	
Preprocedural TIMI flow grade			0.5178
0	643 (69.5)	5,106 (67.6)	
I	99 (10.7)	912 (12.1)	
II	67 (7.2)	597 (7.9)	
III	116 (12.5)	934 (12.4)	
Stent implantation	789 (91.1)	6,500 (92.5)	0.1670
Postprocedural TIMI flow grade			0.9833
0	9 (1.0)	68 (1.0)	
I	14 (1.6)	120 (1.7)	
II	21 (2.4)	158 (2.3)	
III	824 (94.9)	6,632 (95.0)	

*PCI, percutaneous coronary intervention; STEMI, ST-segment elevation myocardial infarction; NSTEMI, non-ST-segment elevation myocardial infarction; TIMI, Thrombolysis In Myocardial Infarction. Data are reported as n (%)*.

### In-hospital Management and Outcomes

Compared with patients aged >45 years, young patients were more likely to be treated with PCI (76.9 vs 61.4%, *p* < 0.0001). Medications, including dual antiplatelet therapy, angiotensin converting enzyme inhibitors (ACEI) or angiotensin II receptor blockers (ARB), β blockers, and statins, were more commonly used in patients aged ≤45 years than in older patients at discharge (Table 3).

**Table 3:** In-hospital management and outcomes of patients in different age groups.

**Variables**	**Patients aged ≤45 years**	**Patients aged >45 years**	***P*-value**
	**(*n* = 2,042)**	**(*n* = 22,083)**	
In-hospital PCI	1,551 (76.9)	13,354 (61.4)	<0.0001
Medications at discharge			
Antiplatelet therapy			<0.0001
Dual antiplatelet therapy	1,926 (95.9)	20,169 (93.0)	
Single antiplatelet therapy	67 (3.3)	1163 (5.4)	
None	16 (0.8)	358 (1.7)	
ACEI/ARB	1,223 (61.6)	12,680 (59.1)	0.0320
β blockers	1,580 (79.4)	14,800 (69.0)	<0.0001
Statins	1,819 (97.7)	19,648 (96.9)	0.0431
In-hospital outcomes			
All-cause death	41 (2.0)	1,556 (7.0)	<0.0001
Recurrent myocardial infarction	3 (0.1)	170 (0.8)	0.0001
Stroke	4 (0.2)	192 (0.9)	0.0001
MACCE	45 (2.2)	1,796 (8.1)	<0.0001
Length of stay	9 (7, 13)	10 (7, 14)	0.0563

*PCI, percutaneous coronary intervention; ACEI, angiotensin converting enzyme inhibitor; ARB, angiotensin II receptor blocker; MACCE, major adverse cardiac and cerebrovascular events. Data are reported as n (%) and median (quartiles)*.

The rates of in-hospital death, recurrent myocardial infarction, stroke, and MACCE were significantly lower in patients aged ≤45 years than in older patients (Table 3). After multivariate adjustment, differences of mortality and MACCE remained significant between two groups (adjusted OR for all-cause death: 0.463, 95% CI 0.332–0.647, *p* < 0.0001; adjusted OR for MACCE: 0.423, 95% CI 0.308–0.580, *p* < 0.0001).

### 2-Year Clinical Outcomes

**Figure 2:**
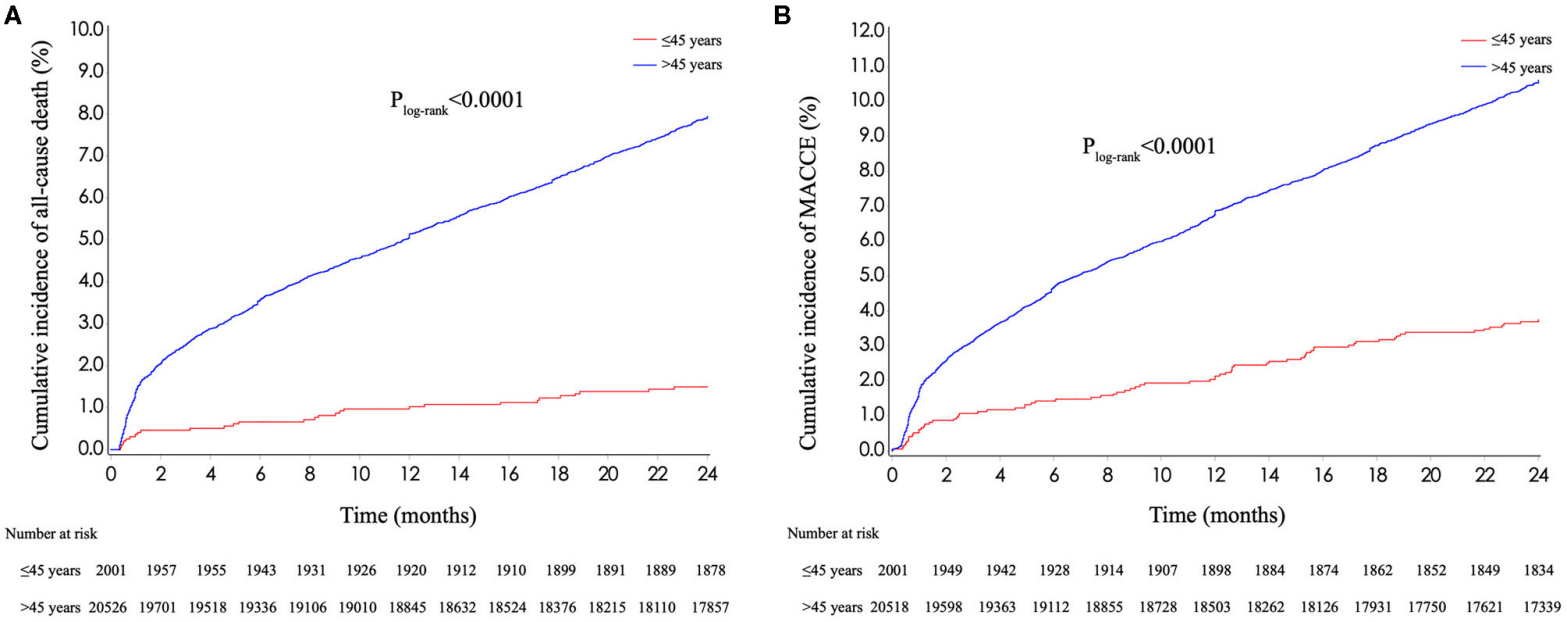
Kaplan–Meier curves stratified by age. **(A)** Kaplan–Meier curves for all-cause death stratified by age. **(B)** Kaplan–Meier curves for MACCE stratified by age. MACCE, major adverse cardiac and cerebrovascular events.

The cumulative rates of all-cause death and MACCE were significantly lower in patients aged ≤45 years than those in older patients (Figure 2; 1.5 vs 7.9% for all-cause death and 3.8 vs 10.6% for MACCE, both *p*_log−rank_ <0.0001). At 2-year follow-up, a smaller proportion of young patients admitted again for heart failure compared with patients aged >45 years (3.0 vs 8.1%, *p* < 0.0001). After multivariate adjustment, young patients had lower risk of all-cause death and MACCE than older patients (adjusted HR for all-cause death: 0.393, 95% CI 0.270–0.571, *p* < 0.0001; adjusted HR for MACCE: 0.603, 95% CI 0.475–0.767, *p* < 0.0001).

From 30-day to 1-year follow-up, use rates of dual antiplatelet therapy, ACEI/ARB, β blockers, and statins decreased from 91.8 to 72.5%, 61.4 to 55.5%, 79.1 to 72.9%, and 95.4 to 89.9% in patients aged ≤45 years, respectively. Dual antiplatelet therapy and β blockers were more frequently used in young patients at 30-day, 6-month, and 1-year follow-up compared with older patients (Table 4).

**Table 4:** Follow-up outcomes of patients in different age groups.

**Variables**	**Patients aged ≤45 years**	**Patients aged >45 years**	***P*-value**
	**(*n* = 2,001)**	**(*n* = 20,527)**	
Adverse events at 2-year follow-up			
All-cause death	29 (1.5)	1,583 (7.9)	<0.0001
Recurrent myocardial infarction	35 (1.8)	406 (2.2)	0.3635
Stroke	11 (0.6)	229 (1.2)	0.0127
MACCE	73 (3.8)	2,112 (10.6)	<0.0001
All-cause readmission	407 (21.3)	6,027 (31.7)	<0.0001
Rehospitalization for heart failure	58 (3.0)	1,536 (8.1)	<0.0001
Medications at 30-day follow-up			
Antiplatelet therapy			<0.0001
Dual antiplatelet therapy	1,508 (91.8)	14,196 (86.9)	
Single antiplatelet therapy	114 (6.9)	1,731 (10.6)	
None	20 (1.2)	413 (2.5)	
ACEI/ARB	992 (61.4)	9,471 (59.0)	0.0665
β blockers	1,285 (79.1)	11,602 (71.5)	<0.0001
Statins	1,565 (95.4)	15,500 (94.9)	0.3163
Medications at 6-month follow-up			
Antiplatelet therapy			<0.0001
Dual antiplatelet therapy	1,297 (86.4)	11,877 (80.3)	
Single antiplatelet therapy	165 (11.0)	2,365 (16.0)	
None	40 (2.7)	554 (3.7)	
ACEI/ARB	860 (58.0)	8,056 (55.4)	0.0584
β blockers	1,111 (74.7)	10,139 (69.1)	<0.0001
Statins	1,399 (93.5)	13,713 (92.7)	0.2803
Medications at 12-month follow-up			
Antiplatelet therapy			<0.0001
Dual antiplatelet therapy	943 (72.5)	8,332 (65.9)	
Single antiplatelet therapy	312 (24.0)	3,700 (29.3)	
None	45 (3.5)	611 (4.8)	
ACEI/ARB	708 (55.5)	6,576 (52.9)	0.0743
β blockers	941 (72.9)	8,545 (68.1)	0.0003
Statins	1,164 (89.9)	11,453 (90.7)	0.3196

*MACCE, major adverse cardiac and cerebrovascular events; ACEI, angiotensin converting enzyme inhibitor; ARB, angiotensin II receptor blocker. Data are reported as n (%)*.

### Predictors of In-hospital and 2-Year Mortality in Young AMI Patients

Variables with *p* < 0.25 were included in multivariate analysis (Supplementary Tables 2, 3). After multivariate analysis, sex, Killip grade, cardiac arrest at admission, LVEF, and leukocyte count were independent predictors of in-hospital death in patients aged ≤45 years (Table 5). Variables associated with 2-year mortality included LVEF, creatinine level, and education level (Table 6). Univariate and multivariate analyses of mortality in patients aged >45 years are presented in Supplementary Tables 4–7. Notably, age was not an independent predictor of in-hospital or 2-year mortality in patients aged ≤45 years.

**Table 5:** Multivariate analysis of in-hospital mortality in young patients.

	**All-cause death**	
	**OR (95% CI)**	***P*-value**
Women (vs. men)	3.263 (1.294, 8.227)	0.0122
Killip grade		
II (vs. I)	0.677 (0.198, 2.315)	0.5341
III (vs. I)	3.071 (0.772, 12,212)	0.1112
IV (vs. I)	6.892 (2.483, 19.132)	0.0002
Cardiac arrest at admission (vs. no)	4.655 (1.303, 16.626)	0.0179
Leukocyte count (per 10^9^/L increase)	1.099 (1.035, 1.168)	0.0021
LVEF (per 1% increase)	0.960 (0.935, 0.986)	0.0027

*LVEF, left ventricular ejection fraction; OR, odds ratio; CI, confidence interval*.

**Table 6:** Multivariate analysis of 2-year mortality in young patients.

	**All-cause death**	
	**HR (95% CI)**	***P*-value**
Education level (vs university/college or higher)		
Secondary school	4.115 (0.547, 30.959)	0.1695
Primary school or illiterate	12.199 (1.540, 96.603)	0.0178
Creatinine level (per 1 μmol/L increase)	1.004 (1.001, 1.007)	0.0163
LVEF (per 1% increase)	0.946 (0.921, 0.972)	<0.0001

*LVEF, left ventricular ejection fraction; HR, hazard ratio; CI, confidence interval*.

### Gender Disparities in Young AMI Patients

Baseline characteristics of young patients stratified by sex are presented in Table 7. Of 2,042 patients in young group, 116 (5.7%) were female. The mean age in two groups were 39.92 ± 4.62 and 40.45 ± 5.03 years, respectively (men vs. women, *p* = 0.2296). Compared with young men, young women were less likely to be current smokers and have university/college degree or above. No significant differences were observed in the prevalence of diabetes mellitus, hypertension, prior angina pectoris, prior myocardial infarction, prior heart failure, prior stroke, and family history of premature coronary artery disease between groups. Symptom onset to admission time of young women was significantly longer than that of young men.

**Table 7:** Baseline characteristics of young patients stratified by sex.

**Variables**	**Men**	**Women**	***P*-value**
	**(*n* = 1,926)**	**(*n* = 116)**	
Patient demographics			
Age, years	39.92 ± 4.62	40.45 ± 5.03	0.2296
Self-paying medications	229 (12.7)	16 (14.5)	0.5717
Education level			<0.0001
University/college or higher	306 (22.2)	10 (11.0)	
Secondary school	813 (59.0)	43 (47.3)	
Primary school or illiterate	258 (18.7)	38 (41.8)	
Living alone	66 (3.5)	3 (2.6)	—
BMI	25.51 ± 3.36	24.27 ± 4.01	0.0018
BMI ≥28 kg/m^2^	350 (18.9)	10 (8.9)	0.0041
Medical history			
Diabetes mellitus	206 (11.2)	14 (12.6)	0.6474
Hypertension	618 (33.2)	44 (38.6)	0.2386
Hyperlipidemia	182 (10.7)	6 (5.6)	0.0686
Current smoker	1435 (75.7)	14 (12.2)	<0.0001
Prior angina pectoris	330 (18.3)	24 (21.6)	0.3973
Prior myocardial infarction	84 (4.6)	3 (2.7)	0.4800
Prior heart failure	15 (0.8)	3 (2.7)	0.0789
Prior stroke	41 (2.2)	6 (5.3)	0.0505
Prior PCI	60 (3.3)	2 (1.8)	0.5798
Prior renal dysfunction	14 (0.8)	0 (0.0)	—
COPD	5 (0.3)	0 (0.0)	—
Family history of premature CAD	113 (6.8)	11 (10.5)	0.1840
Presenting characteristics			
Symptom			—
Yes	1,894 (99.5)	114 (100.0)	
No	9 (0.5)	0 (0.0)	
Trigger factor			0.0876
Yes	473 (27.2)	21 (19.8)	
No	1,269 (72.8)	85 (80.2)	
Symptom onset to admission time			0.0028
<3 h	544 (28.4)	19 (16.4)	
3–6 h	500 (26.1)	24 (20.7)	
6–12 h	297 (15.5)	27 (23.3)	
12–24 h	215 (11.2)	13 (11.2)	
1–7 days	362 (18.9)	33 (28.4)	
Heart rate	80.19 ± 16.41	79.82 ± 16.00	0.8135
Systolic blood pressure	128.62 ± 22.41	125.95 ± 24.75	0.2187
Killip grade			0.0521
I	1,652 (87.2)	90 (79.6)	
II	181 (9.6)	15 (13.3)	
III	28 (1.5)	4 (3.5)	
IV	34 (1.8)	4 (3.5)	
Cardiac arrest at admission	24 (1.3)	1 (0.9)	—
Diagnosis			0.1910
STEMI	1,634 (84.8)	93 (80.2)	
NSTEMI	292 (15.2)	23 (19.8)	
Anterior wall involvement	993 (52.6)	60 (52.2)	0.9346
Laboratory results			
Creatinine, μmol/L	72.00 (62.60, 83.90)	55.30 (45.00, 67.40)	<0.0001
Hemoglobin, g/L	151.10 ± 17.52	123.57 ± 23.89	<0.0001
Leukocyte count, × 10^9^/L	11.55 ± 4.00	11.03 ± 4.10	0.1822
LVEF, %	55.03 ± 10.28	54.52 ± 11.15	0.6344

*BMI, body mass index; PCI, percutaneous coronary intervention; COPD, chronic obstructive pulmonary disease; CAD, coronary artery disease; STEMI, ST-segment elevation myocardial infarction; NSTEMI, non-ST-segment elevation myocardial infarction; LVEF, left ventricular ejection fraction. Data are reported as n (%), mean ± SD or median (quartiles)*.

Among patients aged ≤45 years, 943 (46.2%) underwent primary PCI. Angiographic characteristics are summarized in Supplementary Table 8. Notably, although women had significantly higher preprocedural TIMI flow grade compared with men at the same age, data showed a reversal of constituent ratios after operations. Similar results were observed in young STEMI patients undergoing primary PCI, which are shown in Supplementary Table 9.

**Figure 3:**
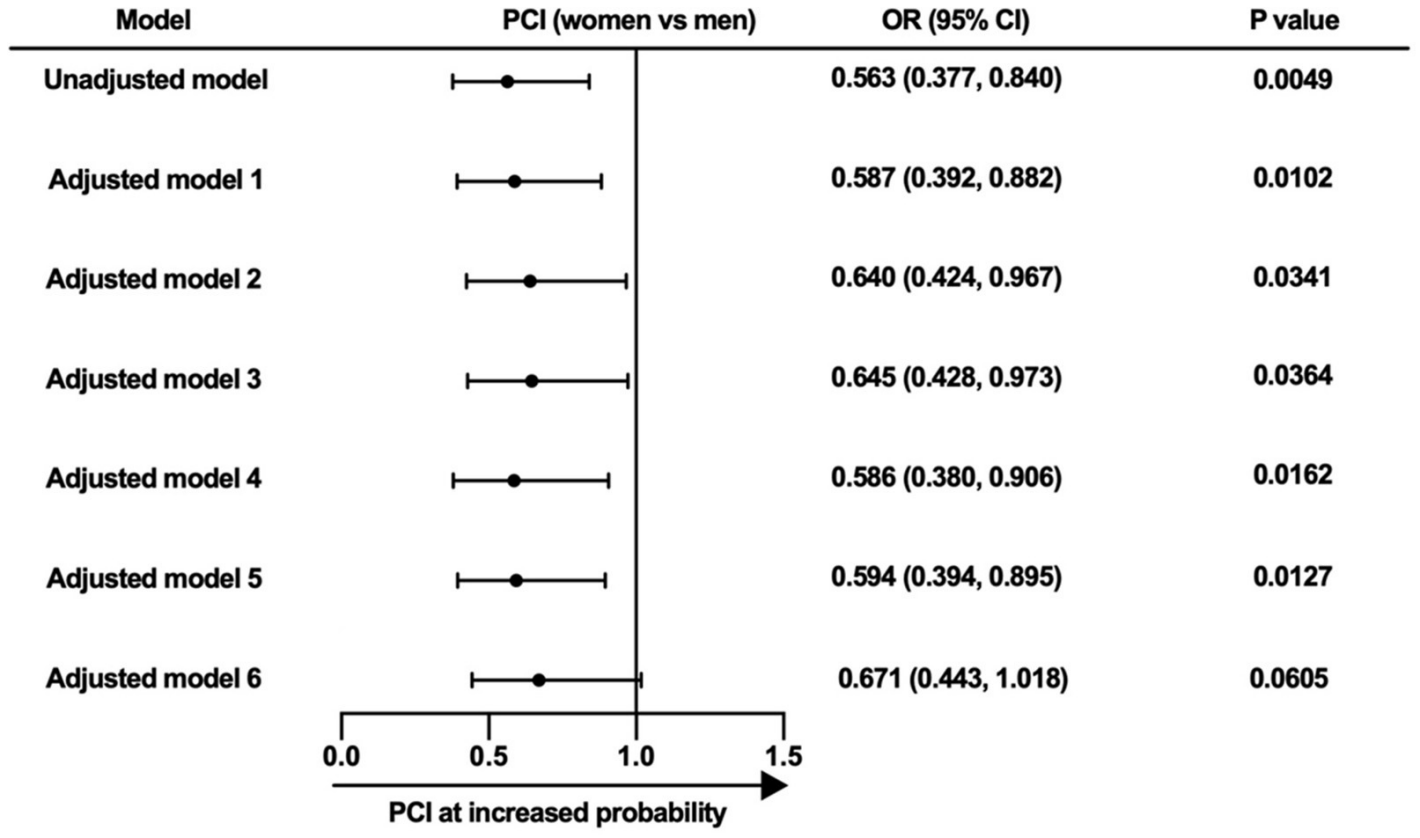
Multivariate analysis for the likelihood of receiving PCI. Model 1, adjusted for symptom onset to admission time. Model 2, adjusted for symptom onset to admission time and education level. Model 3, adjusted for age, medical insurance, education level, living status, and body mass index. Model 4, adjusted for diabetes mellitus, hypertension, hyperlipidemia, smoking status, prior angina pectoris, prior myocardial infarction, prior heart failure, prior stroke, prior PCI, prior renal dysfunction, chronic obstructive pulmonary disease, and family history of premature coronary atherosclerosis heart disease. Model 5, adjusted for heart rate, systolic blood pressure, Killip grade, cardiac arrest at admission, diagnosis, and anterior wall involvement. Model 6, adjusted for age, medical insurance, education level, living status, body mass index, and symptom onset to admission time. PCI, percutaneous coronary intervention; OR, odds ratio; CI, confidence interval.

Compared with men aged ≤45 years, young women were less likely to be treated with PCI (Supplementary Table 10; 66.1 vs 77.6%, *p* = 0.0064). This difference disappeared only after adjustment for patients' demographics and symptom onset to admission time (Figure 3). Dual antiplatelet therapy and ACEI/ARB were also less frequently used at discharge in young women than men at the same age. After multivariate adjustment, young women suffered significantly higher risk of in-hospital death and MACCE than young men (adjusted OR for all-cause death: 5.767, 95% CI 1.580–21.049, *p* = 0.0080; adjusted OR for MACCE: 3.981, 95% CI 1.150–13.784, *p* = 0.0292).

**Figure 4:**
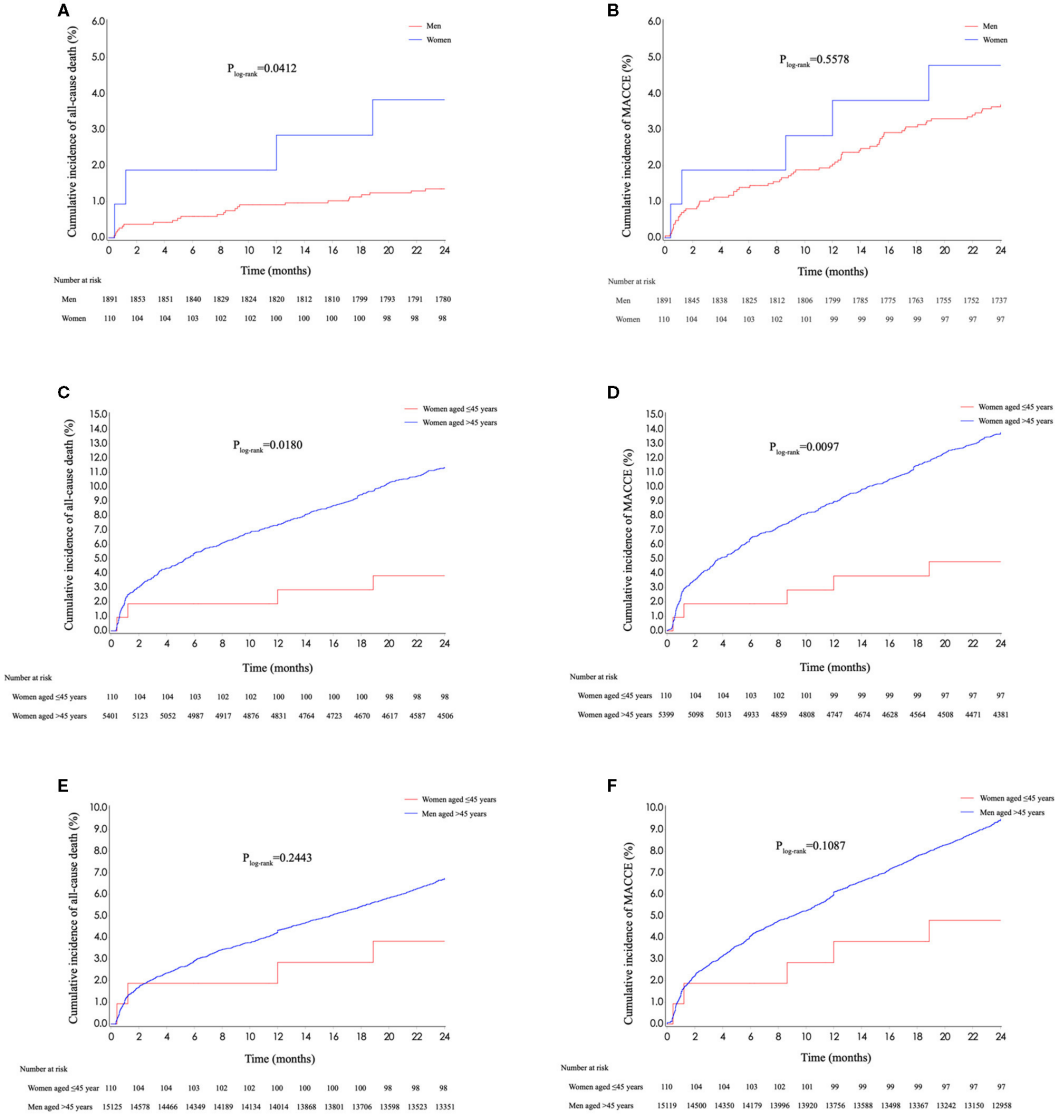
Kaplan–Meier curves stratified by sex and age. **(A)** Kaplan–Meier curves for all-cause death stratified by sex in young patients. **(B)** Kaplan–Meier curves for MACCE stratified by sex in young patients. **(C)** Kaplan–Meier curves for all-cause death stratified by age in women. **(D)** Kaplan–Meier curves for MACCE stratified by age in women. **(E)** Kaplan–Meier curves for all-cause death of young women and older men. **(F)** Kaplan–Meier curves for all-cause death of young women and older men. MACCE, major adverse cardiac and cerebrovascular events.

Women aged ≤45 years experienced a higher cumulative incidence of all-cause death than men at the same age (Figure 4; Supplementary Table 11; 3.8 vs. 1.4%, *p*_log−rank_ = 0.0412). However, this difference disappeared after multivariate adjustment (adjusted HR: 1.739, 95% CI 0.387–7.811, *p* = 0.4703). When comparing 2-year outcomes between young women and older men, no statistical differences were observed in cumulative rates of all-cause death and MACCE (Figure 4; *p*_log−rank_ = 0.2443 for all-cause death; *p*_log−rank_ = 0.1087 for MACCE). The use rates of medications were similar between young women and men, except for small differences of antiplatelet therapy at 6 months and statins at 6- and 12-month follow-up (Supplementary Table 11).

## Discussion

Using data from a large, nationwide, prospective registry, we found patients aged ≤45 years accounted for 8.5% of AMI patients in China. Young patients were more likely to be male and have a medical history of hyperlipidemia. Smoking was the predominant cardiovascular risk factor in patients aged ≤45 years, and 85.2% of them had at least one modifiable risk factor. Young patients were more likely to present with left anterior descending artery related myocardial infarction and be treated with PCI than older patients. In-hospital and long-term outcomes, including 1-year medication use, were significantly better in young patients compared with older patients. Several predictors of all-cause death, such as lower education level and LVEF, were identified in patients aged ≤45 years. Women accounted for 5.7% of young AMI patients, and gender differences were revealed in treatments and in-hospital outcomes between young women and men.

So far, there has been no standard definition of “young” age in patients with AMI. In most studies, the thresholds of age were set at 40–45 years, generally not exceeding 55 years (14–17). According to the criterion of the United Nations (18), which defined over 45 years as older adulthood or above, we chose 45 years as the cutoff of age between young and older patients, and found a non-negligible proportion of AMI patients aged ≤45 years.

Modifiable cardiovascular risk factors have attracted a great deal of attention in research of premature AMI because of its crucial role in secondary prevention after acute coronary event (6, 19). Previous studies reported that over 90% of patients with premature AMI had at least one modifiable risk factor, which mainly included smoking, obesity, dyslipidemia, and hypertension (6, 15, 19). Our study showed a slightly smaller proportion of young patients had one or more modifiable risk factors. However, nearly three quarters of AMI patients aged ≤45 years were current smokers in Chinese patients, while this rate was about 56% in some western countries (6, 15). In view of this fact, a much boarder effort should be taken to protect young adults from smoking.

Consistent with previous studies, patients aged ≤45 years had significantly shorter prehospital delay, higher PCI rate and better in-hospital outcomes than older patients (17, 20). However, we found nearly half of young AMI patients had a symptom onset to admission time of 6 h or more. Considering the small number of asymptomatic patients in the study, this phenomenon may be mainly explained by insufficient awareness of acute coronary events in young adults with cardiovascular risk factors and should be improved in the future.

In this study, patients aged ≤45 years had significantly better 2-year clinical outcomes than older patients, including lower rates of all-cause readmission and rehospitalization for heart failure. Besides, we found the usage rates of antiplatelet therapy and β blockers were higher in young patients compared with those aged >45 years at 30-day, 6-month, and 1-year follow-up. Although a few studies from the YOUNG-MI registry or others reported long-term prognosis of patients with premature AMI (8, 21), readmission rate and follow-up medications were not taken into consideration. Recently, a retrospective analysis from the Dresden Myocardial Infarction Registry assessed follow-up medications in myocardial infarction patients aged ≤ 40 years and showed that they were less frequently treated with diuretics, calcium antagonists, and antidiabetic drugs than older patients in the 1st year (14). However, this work was limited by a small number of young patients (82 young patients at 1-year follow-up).

We identified several independent predictors of in-hospital and 2-year all-cause death in patients aged ≤45 years, which could be used to guide risk stratification in young AMI patients. LVEF was a stable predictor for both in-hospital and 2-year mortality. Prognostic value of education level has been highlighted in patients hospitalized with AMI (22). Our work further proved that lower education level was independently associated with 2-year mortality in patients aged ≤45 years. Patients with premature AMI had longer life expectancy and accordingly required longer post-discharge management than older patients. Therefore, education level, which had close relationship with access to medical services (23), was an important associated factor of long-term mortality. In accordance with result from the YOUNG-MI registry (8), increasing age was not associated with higher mortality in young patients after multivariate adjustment, although it was a major index of numerous risk prediction models (24–26).

In this study, the proportion of women in young AMI patients (5.7%) was smaller than about 20% of patients under 40–50 years old in Germany, Norway, and the USA (14, 15, 27), but similar with that in other Asian populations (28, 29). We supposed this phenomenon might be largely driven by racial and cultural differences between Asia and the West. It is plausible that prehospital delay was the main reason for higher no-reflow or slow-flow rates in young women compared with those in young men, according to a previous study in AMI patients of all ages (30). Limited by a small number of young women with TIMI flow grade < III in this study, we could not use multivariate analysis to adjust variables including symptom onset to admission time for further investigation. However, we did conduct multiple adjusted models and found the difference of PCI rates between young women and men might be explained by patients' demographics and symptom onset to admission time, instead of medical history or clinical severity.

Our study showed that young women with AMI had significantly higher risk of in-hospital death than men at the same age even after multivariate adjustment. This result was markedly different with data from the YOUNG-MI registry (27), but in line with results from the Guidelines-Coronary Artery Disease (GWTG-CAD) registry and the China PEACE-Retrospective AMI study (17, 31). Sex differences of in-hospital outcomes in patients with acute coronary syndromes have been a long debating topic indeed. Some explained that higher in-hospital mortality of women should be attributed to serious clinical profiles and insufficient treatments (32). In this study, medical history and presenting characteristics were mostly similar between young women and men, and we performed multivariate analysis. One possible explanation of our finding is that young women might suffer a longer period to the initial treatment after admission compared with men at the same age. Indeed, a previous study has indicated that women had lower peak biomarker levels and physicians were less likely to consider women's AMI symptoms as cardiac origin (16). A relatively long process before establishing diagnosis means unnecessary in-hospital delay of medications including antiplatelet therapy and PCI. These gaps in AMI treatments should be emphasized and improved in young women. When evaluating 2-year clinical outcomes, our study showed women aged ≤45 years had similar 2-year cumulative rates of adverse events with men at older age. Although differences of 2-year mortality between young women and men disappeared after multivariate adjustment, these results suggested young women with AMI as a high-risk group waiting for further investigation and better management.

Our work provided several novel supplements to existing studies on patients with premature AMI. To our best knowledge, it is the largest study in an Asian population focusing on clinical characteristics and prognosis of young AMI patients. The proportion and gender distribution of young patients in this cohort were different from those in western countries (15, 17). In the analyses of clinical outcomes, we reported dynamic change of major medication use for secondary prevention and readmission rate of young AMI patients after discharge. We further identified independent associated factors of both in-hospital and 2-year mortality for young patients, some of which such as education level and LVEF were not included in main prognostic models or shown by previous study on young patients (27), and might facilitate risk assessment in patients with premature AMI. Compared with other work about gender differences in young patients (17, 27), we gave more detailed analyses for differences in rates of PCI, and found significant gender disparities in both in-hospital and post-discharge clinical outcomes.

### Limitations

There are several important limitations in this study. As a retrospective analysis of a prospective cohort, our work was restricted to population and variables which were preset in the CAMI registry. Patients died before admission were not included in this study. Some cardiovascular risk factors, such as substance abuse, were not taken into account. Previous studies have shown that cocaine and/or marijuana use was not rare in myocardial infarction patients aged ≤ 50 years, and was associated with higher mortality (33). However, considering the powerful anti-drug system and very small percentage of drug addicts in China (34), substance abuse may not be a major risk factor or predictor of adverse events in this cohort. Status of risk factor control after discharge was not included in the CAMI registry, so we cannot evaluate its impact on long-term prognosis of young patients with AMI. In addition, women only made up a relatively small proportion of young patients in this cohort. Thus, predictors of in-hospital or 2-year all-cause death were not obtained in young patients stratified by sex. Finally, although covariates were adjusted in multivariate regression models in this observational study, other confounders might exist and lead to wrong conclusions.

## Conclusion

Patients at or under 45 years old were not rare in AMI patients of all ages. Over eight of 10 young patients had at least one modifiable cardiovascular risk factor. Smoking was the most prevalent risk factor in China currently. Young patients received more intensive treatments and had better in-hospital and 2-year clinical outcomes. Several variables which were not included in frequently used risk assessment tools, including LVEF and educational level, could predict in-hospital or long-term mortality in patients aged ≤45 years. There were significant gender disparities in managements and outcomes among young patients with AMI. Young women suffered longer prehospital delay, more insufficient managements, and higher risk of mortality compared with men at the same age, which deserved more attention and should be improved in the future.

## Data Availability Statement

The raw data supporting the conclusions of this article will be made available by the authors, without undue reservation.

## Ethics Statement

The studies involving human participants were reviewed and approved by institutional review board central committee (Fuwai Hospital, Beijing, China). The patients/participants provided their written informed consent to participate in this study.

## Author Contributions

HX: conception, design, and revision. YYa: conception and design. JL: analyzing data and drafting article. XG, JY, XZ, YYe, QD, RF, HS, and XY: analysis and interpretation of data. LN, KL, YZ, and YW: statistical analysis. All authors approved this article and its submission.

## Conflict of Interest

The authors declare that the research was conducted in the absence of any commercial or financial relationships that could be construed as a potential conflict of interest.

## Publisher's Note

All claims expressed in this article are solely those of the authors and do not necessarily represent those of their affiliated organizations, or those of the publisher, the editors and the reviewers. Any product that may be evaluated in this article, or claim that may be made by its manufacturer, is not guaranteed or endorsed by the publisher.
