# Supplementary material for: Clinical Characteristics, Prognosis, and Gender Disparities in Young Patients With Acute Myocardial Infarction
Source: Front Cardiovasc Med. 2021 Aug 12;8:720378. doi: 10.3389/fcvm.2021.720378 (PMC8387867; doi:10.3389/fcvm.2021.720378)
Supplement: Supplementary file 1 [file Data_Sheet_1.docx]

***Supplementary Material***

1. **Supplementary Methods**

To explain the differences in rates of PCI between men and women, six logistic models were conducted and adjusted as follows: model 1 for symptom onset to admission time; model 2 for symptom onset to admission time and education level; model 3 for age, medical insurance, education level, living status and BMI; model 4 for diabetes mellitus, hypertension, hyperlipidemia, smoking status, prior angina pectoris, prior myocardial infarction, prior heart failure, prior stroke, prior PCI, prior renal dysfunction, COPD and family history of premature coronary atherosclerosis heart disease; model 5 for heart rate, systolic blood pressure, Killip grade, cardiac arrest at admission, diagnosis and anterior wall involvement; and model 6 for age, medical insurance, education level, living status, BMI and symptom onset to admission time.

1. **Supplementary Table Legends**

**Supplementary Table 1.** Angiographic characteristics of STEMI patients undergoing primary PCI in different age groups

**Supplementary Table 2.** Univariate analysis of in-hospital mortality in young patients

**Supplementary Table 3.** Univariate analysis of 2-year mortality in young patients

**Supplementary Table 4.** Univariate analysis of in-hospital mortality in patients aged >45 years

**Supplementary Table 5.** Univariate analysis of 2-year mortality in patients aged >45 years

**Supplementary Table 6.** Multivariate analysis of in-hospital mortality in patients aged >45 years

**Supplementary Table 7.** Multivariate analysis of 2-year mortality in patients aged >45 years

**Supplementary Table 8.** Angiographic characteristics of young patients undergoing primary PCI stratified by sex

**Supplementary Table 9.** Angiographic characteristics of young STEMI patients undergoing primary PCI stratified by sex

**Supplementary Table 10.** In-hospital management and outcomes of young patients stratified by sex

**Supplementary Table 11.** Follow-up outcomes of young patients stratified by sex

**Supplementary Table 1. Angiographic characteristics of STEMI patients undergoing primary PCI in different age groups**

| **Variables** | **Patients characterisaged ≤45 years**  **(n=903)** | **Patients aged >45 years**  **(n=7233)** | **P value** |
| --- | --- | --- | --- |
| Transradial approach | 800 (90.9) | 6103 (86.9) | 0.0004 |
| Culprit artery  Left anterior descending artery  Left circumflex artery  Right coronary artery  Left main coronary artery  Others | 496 (56.4)  85 (9.7)  280 (31.8)  4 (0.5)  15 (1.7) | 3397 (48.5)  723 (10.3)  2673 (38.2)  53 (0.8)  154 (2.2) | 0.0004 |
| Preprocedural TIMI flow grade  0  I  II  III | 622 (70.3)  96 (10.8)  60 (6.8)  107 (12.1) | 4864 (69.1)  831 (11.8)  525 (7.5)  822 (11.7) | 0.7045 |
| Stent implantation | 755 (91.0) | 6082 (92.6) | 0.1110 |
| Postprocedural TIMI flow grade  0  I  II  III | 8 (1.0)  14 (1.7)  21 (2.5)  789 (94.8) | 65 (1.0)  111 (1.7)  151 (2.3)  6194 (95.0) | 0.9857 |

Abbreviations: STEMI, ST-segment elevation myocardial infarction; PCI, percutaneous coronary intervention; TIMI, Thrombolysis In Myocardial Infarction. Data are reported as n (%).

**Supplementary Table 2. Univariate analysis of in-hospital mortality in young patients**

|  | All-cause death | |
| --- | --- | --- |
|  | OR (95% CI) | P value |
| Age (per 1 year increase) | 1.028 (0.956, 1.107) | 0.4554 |
| Women (vs men) | 2.947 (1.214, 7.156) | 0.0169 |
| Self-paying medications (vs no) | 1.019 (0.396, 2.622) | 0.9685 |
| Education level (vs university/college or higher)  primary school or illiterate  Secondary school | 1.286 (0.388, 4.260)  1.332 (0.513, 3.461) | 0.6801  0.5557 |
| Living alone (vs no) | 0.711 (0.096, 5.246) | 0.7384 |
| BMI (per 1kg/m^2^ increase) | 0.950 (0.861, 1.049) | 0.3133 |
| Diabetes mellitus (vs no) | 1.154 (0.448, 2.972) | 0.7670 |
| Hypertension (vs no) | 0.967 (0.498, 1.880) | 0.9221 |
| Hyperlipidemia (vs no) | 1.713 (0.711, 4.128) | 0.2301 |
| Current smoking (vs no) | 1.358 (0.644, 2.862) | 0.4219 |
| Prior angina pectoris (vs no) | 0.981 (0.432, 2.232) | 0.9644 |
| Prior myocardial infarction (vs no) | 1.156 (0.275, 4.867) | 0.8433 |
| Prior heart failure (vs no) | 6.362 (1.414, 28.620) | 0.0159 |
| Prior stroke (vs no) | 1.063 (0.143, 7.896) | 0.9528 |
| Prior PCI (vs no) | — | — |
| Prior renal dysfunction (vs no) | 3.823 (0.488, 29.933) | 0.2015 |
| COPD (vs no) | — | — |
| Family history of premature CAD (vs no) | 2.736 (1.128, 6.633) | 0.0260 |
| Symptom onset to admission time (vs <3h)  3-6h  6-12h  12-24h  1-7 d | 1.589 (0.673, 3.748)  1.178 (0.416, 3.340)  1.688 (0.594, 4.797)  1.127 (0.416, 3.051) | 0.2905  0.7578  0.3261  0.8146 |
| Heart rate (per 1 beat increase) | 1.028 (1.012, 1.044) | 0.0005 |
| Systolic blood pressure (per 1mmHg increase) | 0.982 (0.967, 0.997) | 0.0182 |
| Killip grade (vs I)  II  III  IV | 1.007 (0.303, 3.350)  6.701 (1.924, 23.342)  17.274 (7.255, 41.133) | 0.9910  0.0028  <0.0001 |
| Cardiac arrest at admission (vs no) | 13.757 (4.892, 38.686) | <0.0001 |
| NSTEMI (vs STEMI) | 1.132 (0.497, 2.576) | 0.7681 |
| Anterior wall involvement (vs no) | 1.370 (0.727, 2.582) | 0.3297 |
| Creatinine (per 1μmol/L increase) | 1.003 (1.000, 1.006) | 0.0311 |
| Hemoglobin (per 1g/L increase) | 0.990 (0.977, 1.003) | 0.1186 |
| Leukocyte count (per 10^9^/L increase) | 1.136 (1.074, 1.203) | <0.0001 |
| LVEF (per 1% increase) | 0.951 (0.929, 0.973) | <0.0001 |

Abbreviations: BMI, body mass index; PCI, percutaneous coronary intervention; COPD, chronic obstructive pulmonary disease; CAD, coronary artery disease; NSTEMI, non-ST-segment elevation myocardial infarction; STEMI, ST-segment elevation myocardial infarction; LVEF, left ventricular ejection fraction; OR, odds ratio; CI, confidence interval.

**Supplementary Table 3. Univariate analysis of 2-year mortality in young patients**

|  | All-cause death | |
| --- | --- | --- |
|  | HR (95% CI) | P value |
| Age (per 1 year increase) | 0.986 (0.914, 1.064) | 0.7140 |
| Women (vs men) | 2.858 (0.995, 8.213) | 0.0512 |
| Self-paying medications (vs no) | 1.590 (0.607, 4.166) | 0.3457 |
| Education level (vs university/college or higher)  primary school or illiterate  Secondary school | 10.800 (1.383, 84.366)  4.020 (0.537, 30.111) | 0.0233  0.1757 |
| Living alone (vs no) | 3.329 (1.008, 11.000) | 0.0485 |
| BMI (per 1kg/m^2^ increase) | 0.941 (0.836, 1.058) | 0.3103 |
| Diabetes mellitus (vs no) | 0.941 (0.285, 3.107) | 0.9199 |
| Hypertension (vs no) | 1.276 (0.603, 2.702) | 0.5237 |
| Hyperlipidemia (vs no) | 1.602 (0.558, 4.604) | 0.3812 |
| Current smoking (vs no) | 0.615 (0.291, 1.303) | 0.2047 |
| Prior angina pectoris (vs no) | 1.496 (0.639, 3.501) | 0.3535 |
| Prior myocardial infarction (vs no) | 0.789 (0.107, 5.795) | 0.8155 |
| Prior heart failure (vs no) | 4.297 (0.585, 31.575) | 0.1519 |
| Prior stroke (vs no) | — | — |
| Prior PCI (vs no) | 1.086 (0.148, 7.982) | 0.9354 |
| Prior renal dysfunction (vs no) | — | — |
| COPD (vs no) | — | — |
| Family history of premature CAD (vs no) | 1.893 (0.573, 6.255) | 0.2952 |
| Symptom onset to admission time (vs <3h)  3-6h  6-12h  12-24h  1-7 d | 1.259 (0.457, 3.472)  1.269 (0.403, 3.999)  0.711 (0.148, 3.422)  1.466 (0.514, 4.179) | 0.6564  0.6839  0.6705  0.4743 |
| Heart rate (per 1 beat increase) | 1.023 (1.005, 1.042) | 0.0127 |
| Systolic blood pressure (per 1mmHg increase) | 0.996 (0.979, 1.013) | 0.6171 |
| Killip grade (vs I)  II  III  IV | 0.787 (0.186, 3.338)  5.293 (1.248, 22.446)  5.152 (1.215, 21.853) | 0.7452  0.0238  0.0262 |
| Cardiac arrest at admission (vs no) | — | — |
| NSTEMI (vs STEMI) | 2.119 (0.939, 4.785) | 0.0706 |
| Anterior wall involvement (vs no) | 0.624 (0.298, 1.306) | 0.2103 |
| Creatinine (per 1μmol/L increase) | 1.004 (1.001, 1.007) | 0.0079 |
| Hemoglobin (per 1g/L increase) | 0.994 (0.977, 1.011) | 0.4768 |
| Leukocyte count (per 10^9^/L increase) | 1.083 (1.004, 1.169) | 0.0404 |
| LVEF (per 1% increase) | 0.953 (0.929, 0.977) | 0.0002 |
| In-hospital PCI (vs no) | 0.461 (0.218, 0.977) | 0.0433 |
| Antiplatelet therapy (vs dual antiplatelet therapy)  Single antiplatelet therapy  None | 1.082 (0.147, 7.960)  10.616 (1.442, 78.134) | 0.9386  0.0204 |
| ACEI/ARB | 1.381 (0.664, 2.870) | 0.3876 |
| β blockers | 0.868 (0.331, 2.274) | 0.7727 |
| Statins | 1.895 (0.258, 13.927) | 0.5298 |

Abbreviations: BMI, body mass index; PCI, percutaneous coronary intervention; COPD, chronic obstructive pulmonary disease; CAD, coronary artery disease; NSTEMI, non-ST-segment elevation myocardial infarction; STEMI, ST-segment elevation myocardial infarction; LVEF, left ventricular ejection fraction; ACEI, angiotensin converting enzyme inhibitor; ARB, angiotensin II receptor blocker; HR, hazard ratio; CI, confidence interval.

**Supplementary Table 4. Univariate analysis of in-hospital mortality in patients aged >45 years**

|  | All-cause death | |
| --- | --- | --- |
|  | OR (95% CI) | P value |
| Age (per 1 year increase) | 1.069 (1.064, 1.075) | <0.0001 |
| Women (vs men) | 2.047 (1.842, 2.274) | <0.0001 |
| Self-paying medications (vs no) | 0.746 (0.597, 0.933) | 0.0103 |
| Education level (vs university/college or higher)  Primary school or illiterate  Secondary school | 1.958 (1.527, 2.510)  1.221 (0.935, 1.593) | <0.0001  0.1421 |
| Living alone (vs no) | 1.303 (0.985, 1.723) | 0.0634 |
| BMI (per 1kg/m^2^ increase) | 0.927 (0.910, 0.944) | <0.0001 |
| Diabetes mellitus (vs no) | 1.206 (1.066, 1.365) | 0.0030 |
| Hypertension (vs no) | 1.125 (1.014, 1.249) | 0.0262 |
| Hyperlipidemia (vs no) | 0.429 (0.321, 0.573) | <0.0001 |
| Current smoking (vs no) | 0.429 (0.381, 0.484) | <0.0001 |
| Prior angina pectoris (vs no) | 1.019 (0.907, 1.144) | 0.7570 |
| Prior myocardial infarction (vs no) | 1.271 (1.059, 1.525) | 0.0098 |
| Prior heart failure (vs no) | 2.932 (2.343, 3.670) | <0.0001 |
| Prior stroke (vs no) | 1.645 (1.418, 1.908) | <0.0001 |
| Prior PCI (vs no) | 0.594 (0.442, 0.800) | 0.0006 |
| Prior renal dysfunction (vs no) | 1.929 (1.367, 2.721) | 0.0002 |
| COPD (vs no) | 2.187 (1.671, 2.863) | <0.0001 |
| Family history of premature CAD (vs no) | 0.377 (0.238, 0.596) | <0.0001 |
| Symptom onset to admission time (vs <3h)  3-6h  6-12h  12-24h  1-7 d | 1.010 (0.854, 1.195)  1.247 (1.044, 1.488)  1.459 (1.208, 1.763)  1.479 (1.270, 1.722) | 0.9048  0.0147  <0.0001  <0.0001 |
| Heart rate (per 1 beat increase) | 1.024 (1.021, 1.026) | <0.0001 |
| Systolic blood pressure (per 1mmHg increase) | 0.980 (0.978, 0.982) | <0.0001 |
| Killip grade (vs I)  II  III  IV | 2.213 (1.938, 2.528)  4.297 (3.609, 5.117)  10.854 (9.289, 12.684) | <0.0001  <0.0001  <0.0001 |
| Cardiac arrest at admission (vs no) | 6.630 (5.077, 8.659) | <0.0001 |
| NSTEMI (vs STEMI) | 0.883 (0.781, 0.997) | 0.0452 |
| Anterior wall involvement (vs no) | 1.358 (1.224, 1.505) | <0.0001 |
| LVEF (per 1% increase) | 0.973 (0.968, 0.977) | <0.0001 |
| Creatinine (per 1μmol/L increase) | 1.0003 (1.0001, 1.0004) | 0.0001 |
| Hemoglobin (per 1g/L increase) | 0.984 (0.982, 0.986) | <0.0001 |
| Leukocyte count (per 10^9^/L increase) | 1.113 (1.100, 1.126) | <0.0001 |

Abbreviations: BMI, body mass index; PCI, percutaneous coronary intervention; COPD, chronic obstructive pulmonary disease; CAD, coronary artery disease; NSTEMI, non-ST-segment elevation myocardial infarction; STEMI, ST-segment elevation myocardial infarction; LVEF, left ventricular ejection fraction; OR, odds ratio; CI, confidence interval.

**Supplementary Table 5. Univariate analysis of 2-year mortality in patients aged >45 years**

|  | All-cause death | |
| --- | --- | --- |
|  | HR (95% CI) | P value |
| Age (per 1 year increase) | 1.082 (1.077, 1.087) | <0.0001 |
| Women (vs men) | 1.737 (1.569, 1.922) | <0.0001 |
| Self-paying medications (vs no) | 0.866 (0.706, 1.063) | 0.1693 |
| Education level (vs university/college or higher)  Primary school or illiterate  Secondary school | 1.843 (1.457, 2.331)  1.326 (1.033, 1.702) | <0.0001  0.0267 |
| Living alone (vs no) | 1.534 (1.197, 1.966) | 0.0007 |
| BMI (per 1kg/m2 increase) | 0.908 (0.892, 0.924) | <0.0001 |
| Diabetes mellitus (vs no) | 1.488 (1.331, 1.665) | <0.0001 |
| Hypertension (vs no) | 1.355 (1.224, 1.499) | <0.0001 |
| Hyperlipidemia (vs no) | 0.738 (0.593, 0.918) | 0.0064 |
| Current smoking (vs no) | 0.482 (0.431, 0.539) | <0.0001 |
| Prior angina pectoris (vs no) | 1.270 (1.142, 1.412) | <0.0001 |
| Prior myocardial infarction (vs no) | 2.002 (1.729, 2.318) | <0.0001 |
| Prior heart failure (vs no) | 5.488 (4.645, 6.484) | <0.0001 |
| Prior stroke (vs no) | 2.039 (1.788, 2.324) | <0.0001 |
| Prior PCI (vs no) | 1.048 (0.840, 1.307) | 0.6785 |
| Prior renal dysfunction (vs no) | 3.577 (2.785, 4.593) | <0.0001 |
| COPD (vs no) | 2.910 (2.327, 3.639) | <0.0001 |
| Family history of premature CAD (vs no) | 0.413 (0.271, 0.629) | <0.0001 |
| Symptom onset to admission time (vs <3h)  3-6h  6-12h  12-24h  1-7 d | 1.131 (0.961, 1.332)  1.273 (1.068, 1.517)  1.465 (1.215, 1.767)  1.734 (1.496, 2.009) | 0.1390  0.0071  <0.0001  <0.0001 |
| Heart rate (per 1 beat increase) | 1.021 (1.019, 1.023) | <0.0001 |
| Systolic blood pressure (per 1mmHg increase) | 1.002 (1.000, 1.004) | 0.0249 |
| Killip grade (vs I)  II  III  IV | 2.449 (2.175, 2.757)  5.464 (4.723, 6.320)  4.731 (3.944, 5.675) | <0.0001  <0.0001  <0.0001 |
| Cardiac arrest at admission (vs no) | 0.985 (0.571, 1.701) | 0.9578 |
| NSTEMI (vs STEMI) | 1.868 (1.688, 2.067) | <0.0001 |
| Anterior wall involvement (vs no) | 1.199 (1.087, 1.323) | 0.0003 |
| LVEF (per 1% increase) | 0.962 (0.958, 0.966) | <0.0001 |
| Creatinine (per 1μmol/L increase) | 1.0002 (1.0001, 1.0002) | <0.0001 |
| Hemoglobin (per 1g/L increase) | 0.983 (0.981, 0.985) | <0.0001 |
| Leukocyte count (per 10^9^/L increase) | 1.041 (1.028, 1.054) | <0.0001 |
| In-hospital PCI (vs no) | 0.221 (0.199, 0.246) | <0.0001 |
| Antiplatelet therapy (vs dual antiplatelet therapy)  Single antiplatelet therapy  None | 2.032 (1.713, 2.411)  2.604 (1.909, 3.553) | <0.0001  <0.0001 |
| ACEI/ARB (vs yes) | 1.244 (1.126, 1.374) | <0.0001 |
| β blockers (vs yes) | 1.522 (1.375, 1.686) | <0.0001 |
| Statins (vs yes) | 1.783 (1.359, 2.339) | <0.0001 |

Abbreviations: BMI, body mass index; PCI, percutaneous coronary intervention; COPD, chronic obstructive pulmonary disease; CAD, coronary artery disease; NSTEMI, non-ST-segment elevation myocardial infarction; STEMI, ST-segment elevation myocardial infarction; LVEF, left ventricular ejection fraction; ACEI, angiotensin converting enzyme inhibitor; ARB, angiotensin II receptor blocker; HR, hazard ratio; CI, confidence interval.

**Supplementary Table 6. Multivariate analysis of in-hospital mortality in patients aged >45 years**

|  | All-cause death | |
| --- | --- | --- |
|  | OR (95% CI) | P value |
| Age (per 1 year increase) | 1.056 (1.050, 1.062) | <0.0001 |
| Women (vs men) | 1.251 (1.103, 1.419) | 0.0005 |
| Self-paying medications (vs no) | 0.761 (0.598, 0.969) | 0.0265 |
| Hyperlipidemia (vs no) | 0.594 (0.437, 0.808) | 0.0009 |
| Current smoking (vs no) | 0.716 (0.622, 0.824) | <0.0001 |
| Prior myocardial infarction (vs no) | 1.318 (1.056, 1.645) | 0.0147 |
| Prior stroke (vs no) | 1.281 (1.087, 1.509) | 0.0031 |
| Prior PCI (vs no) | 0.573 (0.405, 0.811) | 0.0017 |
| Symptom onset to admission time (vs <3h)  3-6h  6-12h  12-24h  1-7 d | 0.933 (0.777, 1.120)  1.141 (0.940, 1.385)  1.275 (1.035, 1.570)  1.137 (0.959, 1.349) | 0.4556  0.1815  0.0223  0.1392 |
| Heart rate (per 1 beat increase) | 1.016 (1.013, 1.018) | <0.0001 |
| Systolic blood pressure (per 1mmHg increase) | 0.984 (0.982, 0.986) | <0.0001 |
| Killip grade (vs I)  II  III  IV | 1.319 (1.141, 1.524)  1.874 (1.540, 2.280)  4.020 (3.360, 4.810) | 0.0002  <0.0001  <0.0001 |
| Cardiac arrest at admission (vs no) | 3.881 (2.831, 5.320) | <0.0001 |
| NSTEMI (vs STEMI) | 0.723 (0.627, 0.833) | <0.0001 |
| Anterior wall involvement (vs no) | 1.178 (1.046, 1.326) | 0.0068 |
| LVEF (per 1% increase) | 0.994 (0.988, 0.999) | 0.0333 |
| Hemoglobin (per 1g/L increase) | 0.993 (0.991, 0.996) | <0.0001 |
| Leukocyte count (per 10^9^/L increase) | 1.078 (1.064, 1.092) | <0.0001 |

Abbreviations: PCI, percutaneous coronary intervention; NSTEMI, non-ST-segment elevation myocardial infarction; STEMI, ST-segment elevation myocardial infarction; LVEF, left ventricular ejection fraction; OR, odds ratio; CI, confidence interval.

**Supplementary Table 7. Multivariate analysis of 2-year mortality in patients aged >45 years**

|  | All-cause death | |
| --- | --- | --- |
|  | HR (95% CI) | P value |
| Age (per 1 year increase) | 1.051 (1.046, 1.057) | <0.0001 |
| BMI (per 1kg/m^2^ increase) | 0.964 (0.948, 0.981) | <0.0001 |
| Diabetes mellitus (vs no) | 1.266 (1.127, 1.423) | <0.0001 |
| Hypertension (vs no) | 1.131 (1.016, 1.258) | 0.0240 |
| Prior myocardial infarction (vs no) | 1.233 (1.058, 1.437) | 0.0075 |
| Prior heart failure (vs no) | 1.527 (1.271, 1.835) | <0.0001 |
| Prior stroke (vs no) | 1.301 (1.136, 1.489) | 0.0001 |
| Heart rate (per 1 beat increase) | 1.008 (1.006, 1.011) | <0.0001 |
| Killip grade (vs I)  II  III  IV | 1.344 (1.188, 1.522)  1.749 (1.489, 2.055)  1.801 (1.482, 2.189) | <0.0001  <0.0001  <0.0001 |
| NSTEMI (vs STEMI) | 1.137 (1.017, 1.272) | 0.0240 |
| Anterior wall involvement (vs no) | 1.144 (1.031, 1.269) | 0.0111 |
| LVEF (per 1% increase) | 0.977 (0.972, 0.981) | <0.0001 |
| Hemoglobin (per 1g/L increase) | 0.994 (0.991, 0.996) | <0.0001 |
| Leukocyte count (per 10^9^/L increase) | 1.035 (1.024, 1.046 ) | <0.0001 |
| In-hospital PCI (vs no) | 0.416 (0.370, 0.468) | <0.0001 |
| ACEI/ARB (vs yes) | 1.182 (1.064, 1.313) | 0.0019 |
| β blockers (vs yes) | 1.192 (1.069, 1.329) | 0.0015 |
| Statins (vs yes) | 1.339 (1.018, 1.760) | 0.0367 |

Abbreviations: BMI, body mass index; NSTEMI, non-ST-segment elevation myocardial infarction; STEMI, ST-segment elevation myocardial infarction; LVEF, left ventricular ejection fraction; PCI, percutaneous coronary intervention; ACEI, angiotensin converting enzyme inhibitor; ARB, angiotensin II receptor blocker; HR, hazard ratio; CI, confidence interval.

**Supplementary Table 8. Angiographic characteristics of young patients undergoing primary PCI stratified by sex**

| **Variables** | **Men**  **(n=902)** | **Women**  **(n=41)** | **P value** |
| --- | --- | --- | --- |
| Diagnosis  STEMI  NSTEMI | 864 (95.8)  38 (4.2) | 39 (95.1)  2 (4.9) | 0.6911 |
| Transradial approach | 772 (90.9) | 34 (91.9) | — |
| Culprit artery  Left anterior descending artery  Left circumflex artery  Right coronary artery  Left main coronary artery  Others | 486 (55.2)  97 (11.0)  277 (31.4)  4 (0.5)  17 (1.9) | 24 (61.5)  2 (5.1)  12 (30.8)  0 (0.0)  1 (2.6) | 0.6470 |
| Preprocedural TIMI flow grade  0  I  II  III | 624 (70.5)  90 (10.2)  63 (7.1)  108 (12.2) | 19 (47.5)  9 (22.5)  4 (10.0)  8 (20.0) | 0.0099 |
| Stent implantation | 759 (91.4) | 30 (83.3) | 0.1239 |
| Postprocedural TIMI flow grade  0  I  II  III | 6 (0.7)  14 (1.7)  19 (2.3)  793 (95.3) | 3 (8.3)  0 (0.0)  2 (5.6)  31 (86.1) | 0.0045 |

Abbreviations: PCI, percutaneous coronary intervention; STEMI, ST-segment elevation myocardial infarction; NSTEMI, non-ST-segment elevation myocardial infarction; TIMI, Thrombolysis In Myocardial Infarction. Data are reported as n (%).

**Supplementary Table 9. Angiographic characteristics of young STEMI patients undergoing primary PCI stratified by sex**

| **Variables** | **Men**  **(n=864)** | **Women**  **(n=39)** | **P value** |
| --- | --- | --- | --- |
| Transradial approach | 766 (90.9) | 34 (91.9) | — |
| Culprit artery  Left anterior descending artery  Left circumflex artery  Right coronary artery  Left main coronary artery  Others | 473 (56.1)  84 (10.0)  268 (31.8)  4 (0.5)  14 (1.7) | 23 (62.2)  1 (2.7)  12 (32.4)  0 (0.0)  1 (2.7) | 0.4822 |
| Preprocedural TIMI flow grade  0  I  II  III | 605 (71.4)  87 (10.3)  56 (6.6)  99 (11.7) | 17 (44.7)  9 (23.7)  4 (10.5)  8 (21.1) | 0.0029 |
| Stent implantation | 726 (91.2) | 29 (85.3) | 0.2227 |
| Postprocedural TIMI flow grade  0  I  II  III | 5 (0.6)  14 (1.8)  19 (2.4)  760 (95.2) | 3 (8.8)  0 (0.0)  2 (5.9)  29 (85.3) | 0.0034 |

Abbreviations: STEMI, ST-segment elevation myocardial infarction; PCI, percutaneous coronary intervention; TIMI, Thrombolysis In Myocardial Infarction. Data are reported as n (%).

**Supplementary Table 10. In-hospital management and outcomes of young patients stratified by sex**

| **Variables** | **Men**  **(n=1926)** | **Women**  **(n=116)** | **P value** |
| --- | --- | --- | --- |
| In-hospital PCI | 1475 (77.6) | 76 (66.1) | 0.0064 |
| Medications at discharge  Antiplatelet therapy  Dual antiplatelet therapy  Single antiplatelet therapy  None  ACEI/ARB  β blockers  Statins | 1821 (96.1)  58 (3.1)  15 (0.8)  1166 (62.3)  1493 (79.6)  1714 (97.9) | 105 (91.3)  9 (7.8)  1 (0.9)  57 (50.0)  87 (75.7)  105 (95.5) | 0.0239  0.0095  0.3158  0.0980 |
| In-hospital outcomes  All-cause death  Recurrent myocardial infarction  Stroke  MACCE | 35 (1.8)  3 (0.2)  4 (0.2)  39 (2.0) | 6 (5.2)  0 (0.0)  0 (0.0)  6 (5.2) | 0.0259  —  —  0.0391 |
| Length of stay | 9 (7, 13) | 10 (8, 14) | 0.1262 |

Abbreviations: PCI, percutaneous coronary intervention; ACEI, angiotensin converting enzyme inhibitor; ARB, angiotensin II receptor blocker; MACCE, major adverse cardiac and cerebrovascular events. Data are reported as n (%).

**Supplementary Table 11. Follow-up outcomes of young patients stratified by sex**

| **Variables** | **Men**  **(n=1891)** | **Women**  **(n=110)** | **P value** |
| --- | --- | --- | --- |
| Adverse events at 2-year follow-up  All-cause death  Recurrent myocardial infarction  Stroke  MACCE  All-cause readmission  Rehospitalization for heart failure | 25 (1.4)  34 (1.9)  11 (0.6)  68 (3.7)  385 (21.3)  54 (3.0) | 4 (3.8)  1 (1.0)  0 (0.0)  5 (4.8)  22 (22.0)  4 (4.0) | 0.0412  0.5198  0.4339  0.5578  0.8673  0.5434 |
| Medications at 30-day follow-up  Antiplatelet therapy  Dual antiplatelet therapy  Single antiplatelet therapy  None  ACEI/ARB  β blockers  Statins | 1427 (92.1)  105 (6.8)  18 (1.2)  942 (61.8)  1219 (79.5)  1480 (95.6) | 81 (88.0)  9 (9.8)  2 (2.2)  50 (54.3)  66 (72.5)  85 (92.4) | 0.4136  0.1570  0.1257  0.1898 |
| Medications at 6-month follow-up  Antiplatelet therapy  Dual antiplatelet therapy  Single antiplatelet therapy  None  ACEI/ARB  β blockers  Statins | 1236 (86.9)  152 (10.7)  34 (2.4)  822 (58.6)  1055 (75.0)  1329 (93.8) | 61 (76.3)  13 (16.3)  6 (7.5)  38 (47.5)  56 (70.0)  70 (87.5) | 0.0183  0.0523  0.3272  0.0459 |
| Medications at 12-month follow-up  Antiplatelet therapy  Dual antiplatelet therapy  Single antiplatelet therapy  None  ACEI/ARB  β blockers  Statins | 902 (73.3)  287 (23.3)  42 (3.4)  673 (55.8)  891 (73.0)  1109 (90.5) | 41 (59.4)  25 (36.2)  3 (4.3)  35 (51.5)  50 (72.5)  55 (79.7) | 0.0511  0.4898  0.9263  0.0095 |

Abbreviations: MACCE, major adverse cardiac and cerebrovascular events; ACEI, angiotensin converting enzyme inhibitor; ARB, angiotensin II receptor blocker. Data are reported as n (%).
